# Genome-Wide Survey and Development of the First Microsatellite Markers Database (*AnCorDB*) in *Anemone coronaria* L.

**DOI:** 10.3390/ijms23063126

**Published:** 2022-03-14

**Authors:** Matteo Martina, Alberto Acquadro, Lorenzo Barchi, Davide Gulino, Fabio Brusco, Mario Rabaglio, Flavio Portis, Ezio Portis, Sergio Lanteri

**Affiliations:** 1 DISAFA, Plant Genetics and Breeding, University of Turin, Largo P. Braccini 2, 10095 Grugliasco, Italy; matteo.martina@unito.it (M.M.); alberto.acquadro@unito.it (A.A.); lorenzo.barchi@unito.it (L.B.); davide.gulino@unito.it (D.G.); sergio.lanteri@unito.it (S.L.); 2 Biancheri Creazioni, 18033 Camporosso, Italy; fabio@bianchericreazioni.it (F.B.); mario@bianchericreazioni.it (M.R.); 3 Yebokey, 10100 Turin, Italy; flavioportis@gmail.com

**Keywords:** poppy anemone, SSRs, genome sequencing, fingerprinting

## Abstract

*Anemone coronaria* L. (2n = 2x = 16) is a perennial, allogamous, highly heterozygous plant marketed as a cut flower or in gardens. Due to its large genome size, limited efforts have been made in order to develop species-specific molecular markers. We obtained the first draft genome of the species by Illumina sequencing an androgenetic haploid plant of the commercial line “MISTRAL^®^ Magenta”. The genome assembly was obtained by applying the MEGAHIT pipeline and consisted of 2 × 10^6^ scaffolds. The SciRoKo SSR (Simple Sequence Repeats)-search module identified 401.822 perfect and 188.987 imperfect microsatellites motifs. Following, we developed a user-friendly “*Anemone coronaria* Microsatellite DataBase” (*AnCorDB*), which incorporates the Primer3 script, making it possible to design couples of primers for downstream application of the identified SSR markers. Eight genotypes belonging to eight cultivars were used to validate 62 SSRs and a subset of markers was applied for fingerprinting each cultivar, as well as to assess their intra-cultivar variability. The newly developed microsatellite markers will find application in Breeding Rights disputes, developing genetic maps, marker assisted breeding (MAS) strategies, as well as phylogenetic studies.

## 1. Introduction

*Anemone* genus belongs to the *Ranunculaceae* family and the nowadays most cultivated species (*A. coronaria* L., *A. hortensis* L., and *A. pavoniana* Lam.) originated in the Mediterranean basin. *A. coronaria* L., also known as poppy anemone, is an herbaceous, perennial crop cultivated both as a cut-flower and garden plant [1]. It is a diploid species characterized by 16 chromosomes (2n = 2x = 16), but some of the commercial varieties are tetraploid. The cultivars exploited as cut-flower are early flowering and produce robust stems carrying flowers with large petals and sepals, while garden cultivars produce erect leaves and a higher number of smaller flowers with short petioles [2]. Poppy anemone is allogamous, due to protogyny, and highly heterozygous [3]. Although self-pollination is possible, the species is characterized by marked inbreeding depression [4], which precludes the obtainment of pure lines suitable for the production of F1 hybrid seeds. Commercial cultivars are produced by inter-crossing of selected heterozygous plants and show variable levels of internal genetic variability. Growers plant rhizomes, which are generated after one season of nursery cultivation [5]. 

In previous studies, DNA markers techniques have been applied and adapted mainly for access intra-cultivars genetic variability or to perform varietal fingerprinting, refs. [5,6,7] but no examples are reported in literature on the development of DNA species-specific markers. 

The advances in next-generation sequencing (NGS) techniques and the progressive reduction of sequencing costs facilitated the obtainment of draft sequence genomes in many plant species. However, due to the large size of *A. coronaria* genome, estimated between 9.08 and 11.93 Gb according to the analyzed genotype [8,9], a reference genomic sequence of the species is not available. 

We generated the first draft for genome sequence of *A. coronaria*, and we report on the massive microsatellite loci identification following its genome-wide survey. Microsatellite—alias simple sequence repeats (SSR)-markers, are co-dominantly inherited, ubiquitous, highly polymorphic, and have found large application in plant breeding and phylogenetic studies because of their simple application through conventional PCR protocols [10,11,12,13,14,15,16]. Unlike single nucleotide polymorphisms (SNPs), which have become the gold standard among molecular markers, SSRs show the advantage of being multi-allelic and highly informative, characterized by a certain level of transferability between related specie [17,18,19,20], and are easily and automatically scorable. 

Based on the microsatellites identified, we developed a public dynamic database, which also provides need-based primer designing facilities and represents the first on-line SSR loci resource available for the scientific community and breeders of poppy anemone and related species. Furthermore, a set of the newly developed markers have been validated in commercial cultivars. 

## 2. Results and Discussion

### 2.1. Draft Genome Assembly and Annotation

Since *A. coronaria* is a highly heterozygous species, the sequence divergence between alleles in a diploid genotype may hinder a reliable contig assembly of its genome sequence [21,22]. In order to overcome this hurdle, we performed DNA sequencing of a haploid androgenetic plant originated through “in vitro” anther culture of a diploid plant of the cultivar MISTRAL^®^ Magenta. Overall, 91.24 Gb of cleaned reads were generated and used as input for genome assembly (Supplementary Materials). The obtained draft assembly consisted of ~4.7 × 10^6^ scaffolds (N50 = 5046 bp) for a total genome size of 6.94 Gb. By removing scaffolds shorter than 500 bp, their number was reduced to 2 × 10^6^ (N50 = 6157 bp), for a total genome size of ~6.13 Gb (Supplementary Materials). K-mer analyses of Illumina sequencing data were performed in order to estimate the genome size of the MISTRAL^®^ Magenta genotype. For the 19-mer frequency distribution, the number of K-mers was 3,100,416,21, with a plot peak around 4 (times each 19-mear occurs—see Supplementary Materials). According to our analysis (see Section 3), the MISTRAL^®^ Magenta genome size was estimated around 7.8 Gb, leading our final assembly to cover ~78.6% of the genotype genome.

After the masking of the draft genome, about 75% of the sequences were classified as repetitive elements. This result is in accordance with what was previously reported in the literature, namely that the expansion of gigantic genomes has been driven by the proliferation of transposable elements [23,24]. Indeed, also due to the sequencing of short-libraries (270 bp), the huge amount of repetitive content hampered the assembly procedures and biased some assembly metrics. 

The masked assembled draft genome was structurally annotated with the Maker-P suite, identifying an overall number of 26,260 genes (AED ≤ 0.4) covering ~56.12 Mb (0.92%) of the estimated genome size. Functional annotation performed through InterProScan domain inspection highlighted about 84% of the predicted proteins with at least one IPR domain. Among the top SUPERFAMILY domains, the most abundant (8.62%) was SSF56112 (protein kinase-like domain), which acts on regulatory and signaling processes in the eukaryotic cell. The second most represented superfamily (6.19%) was SSF52540 (P-loop containing nucleoside triphosphate hydrolase) which is involved in several UniPathways, such as chlorophyll or CoA biosynthesis, followed by SSF48264 (4.10%-Cytochrome P450). These superfamilies have been previously reported as highly abundant in various genomic backgrounds [25,26,27,28,29].

### 2.2. The SSR Content of the Poppy Anemone Draft Genome

In the assembled poppy anemone genome, a total of 401,822 perfect SSR motifs (density of 65.52 SSR/Mb), which included 42,111 compound SSRs, and 188,987 imperfect SSR motifs were identified (Table 1).

**Figure 1:**
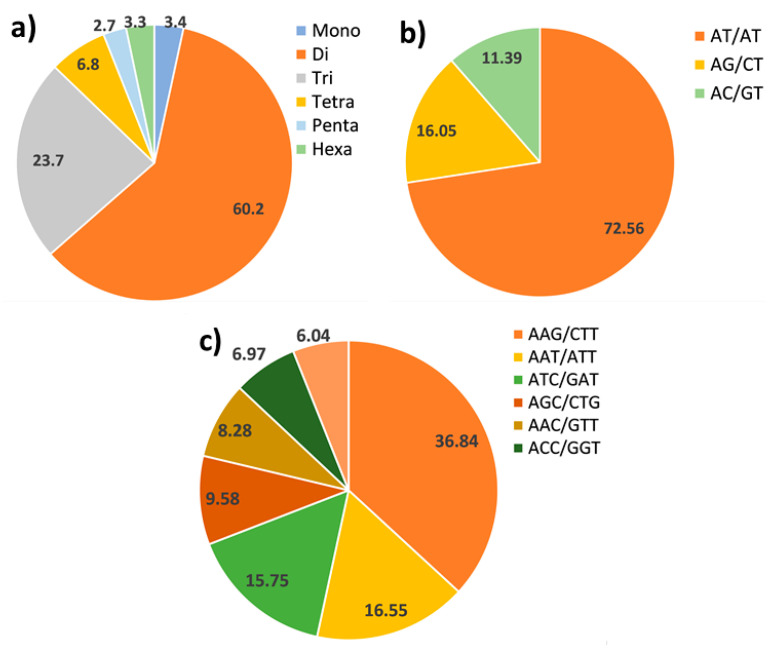
Microsatellites distribution in the poppy anemone genome. (**a**) Percentage distribution of the most frequent classes of SSRs; (**b**) dinucleotides motifs e and (**c**) main trinucleotides motifs identified by SciRoKo.

Six classes of perfect SSRs were evaluated (from mono- to hexanucleotide) for their abundance in the assembled genome. Dinucleotides were the most abundant, in accordance with what has been previously reported in literature [30,31,32,33,34,35,36,37,38,39], representing 60.2% of the identified SSRs. Trinucleotides were the second most abundant class (23.7%), followed by tetranucleotides (6.8%). Penta-, hexa-, and mononucleotides covered the remaining percentage and showed analogous frequency ranging from 2.7 to 3.4% (Figure 1a). The most represented dinucleotide motifs, AT/AT, AG/CT, and AC/GT, accounted respectively for 72.56%, 16.05%, and 11.39% (Figure 1b), while CG/GC motifs were approximately absents (0.009%). The high abundance of AT/AT motifs was in line with a number of previously reported genome surveys, confirming these microsatellites as the most represented dinucleotide motifs in higher plants. Within the trinucleotide repeat motifs, the most abundant were AAG/CTT, accounting for 36.84%, AAT/ATT for 16.55%, and ATC/GAT for 15.75% (Figure 1c).

**Figure 2:**
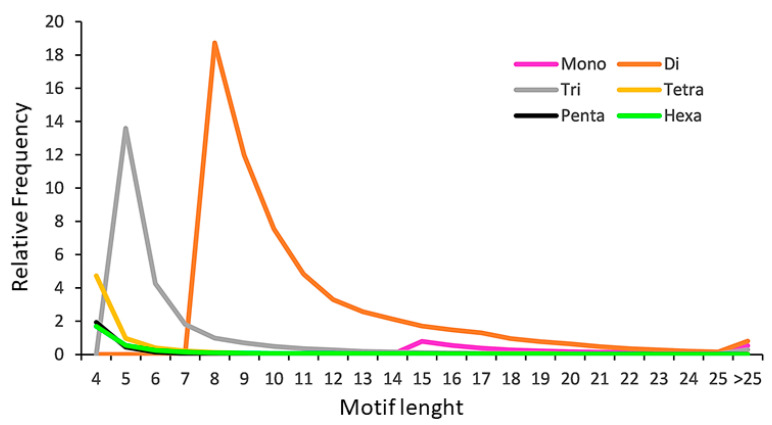
The relative frequency of SSR motifs with different lengths, classified by the number of repeats.

The variation of perfect microsatellites repeats was investigated in all SRR classes (Supplementary Materials). As previously reported, longer repeats (>25) tend to be less abundant in the genome [37,38,40,41]. As can be observed in Figure 2, the tri-, tetra-, penta-, and hexanucleotides relative distribution was higher between one and 10 motif repeats, while mononucleotides distribution increased from 14 motif repeats onward and dinucleotides showed higher abundance between 8 and 19 motif repeats.

**Figure 3:**
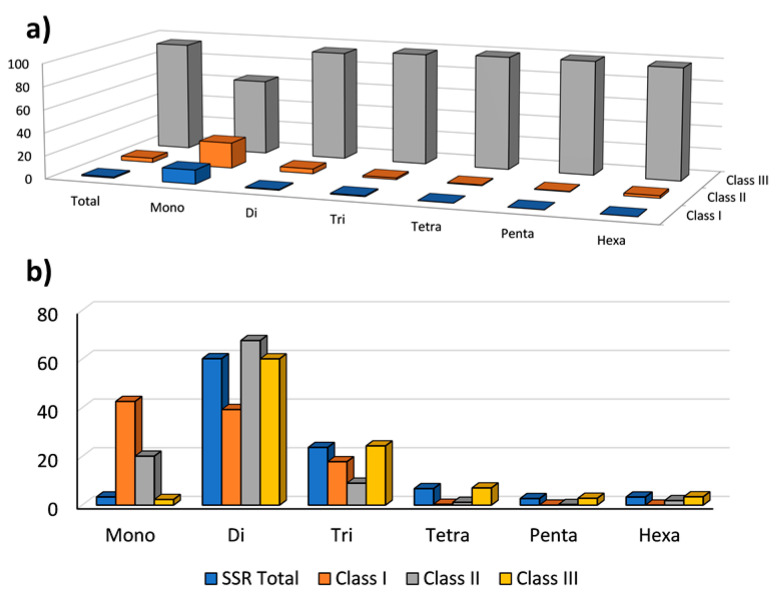
(**a**) The frequency of repeat classes (class I > 30 motif repeats, class II 20–30 motif repeats, class III < 20 motif repeats; (**b**) the distribution of motif type within each class.

Based on the number of motif repeats, 0.95% of SSRs were classified within the hypervariable class I (≥30 motif repeats), 3.71% were assigned to the potentially variable class II (20–30 motif repeats) types, while the remaining 95.34% were included in the variable class III (<20 motif repeats) types (Figure 3a).

Compared with our previously published data [37,38], in which SSR classification was based on microsatellite length (nt), the present classification reports a lower number of SSRs belonging to class I and II. The choice of shifting from a microsatellite length (nt) classification to a repeat number-based one was performed in order to maximize the polymorphism discrimination power and informativeness of the Class I and Class II markers (Figure 3b).

### 2.3. Gene Context of SSRs

The obtained gene annotation made it possible to investigate the distribution of microsatellites across the gene space. Overall, 3223 perfect (0.80% of the total) and 1261 imperfect SSRs (0.67%) were associated with 3223 and 1261 genes respectively, representing 0.23% of the gene space. These SSRs were estimated to cover a total of 134 Kb, values which translates to a density across the gene space of 57.48 and 22.52 SSRs/Mbp for perfect and imperfect motifs, respectively.

**Figure 4:**
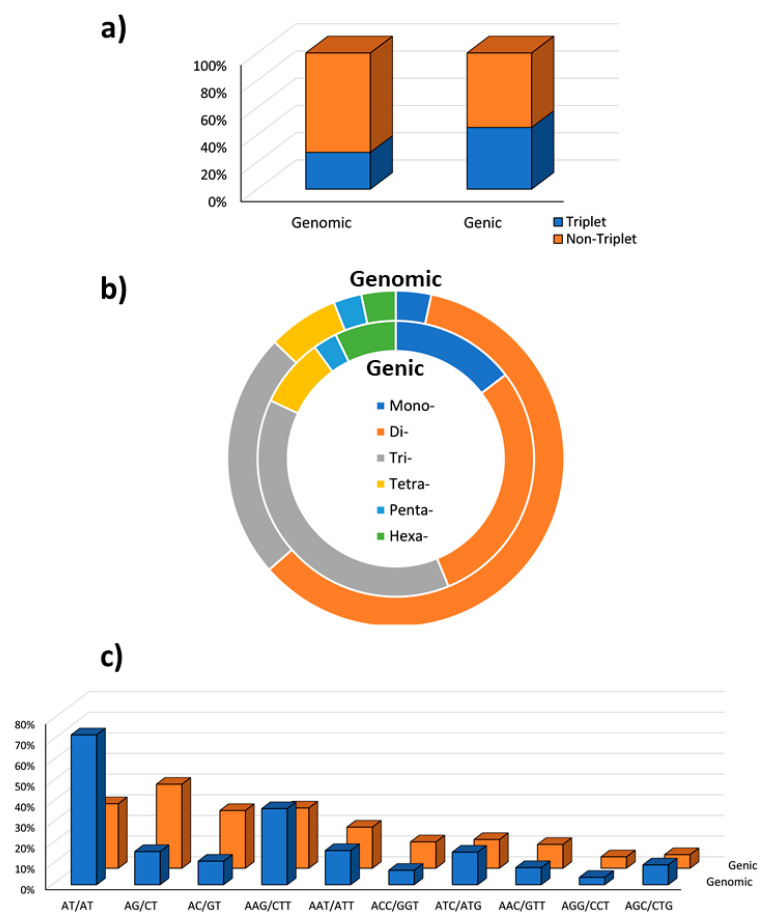
(**a**) Non-triplet SSR vs. triplets SSR; (**b**) distribution of repeat types within perfect and imperfect SSR motifs in both the genomic and genic regions; (**c**) Comparison between di- and trinucleotide repeats in both full genomic regions and gene space.

We also investigated the perfect SSR motifs detected in the global set of genomic and genic SSRs. The microsatellites were classified in non-triplet repeats (mono-, di-, tetra- and pentanucleotides), and triplet repeats (tri- and hexanucleotides), and a fair balance between the two classes (45.33% triplets; 54.67% non-triplets; Figure 4a) was detected in genic SSRs, while in the whole genomic set the triplets were just about 27%.

Trinucleotides were the most common class among the genic perfect microsatellites (38.2%), and the second most common class were the dinucleotides (29.0%; Figure 4b). The predominance of trinucleotides in the gene space has been widely reported in literature as a direct effect of negative selection against frameshift mutations in coding regions [38,42,43,44,45]. Furthermore, the increase of trinucleotides frequency in genomic coding regions might be due to a positive selection for specific single amino-acids [46,47]. For this reason, the most frequent trinucleotides genic perfect SSR motif types were investigated (Figure 4c), identifying AAG/CTT, coding for lysine, as the most represented motif (11.14%), followed by AAT/ATT (7.60%), ATC/GAT (5.31%), and ACC/GGT (4.87%) coding for aspargine, isoleucine, and threonine respectively. In the genic regions, the most common dinucleotides were AG/CT (11.88%) followed by AT/AT (9.12%). The predominance of AG/CT motif in gene sequences has been widely reported in literature, as well as the higher frequency of AT/AT in the non-transcribed regions. Being present in transcripts, genic SSRs have been reported as an important class of “functional markers” (DNA markers derived from functionally characterized sequence motifs [48]) playing a crucial role in gene expression in both mammals and plants [49,50,51,52,53]. Furthermore, genic microsatellite markers have been reported to possess higher portability among related species, making it possible to use them as anchor markers in comparative genetics [54].

**Figure 5:**
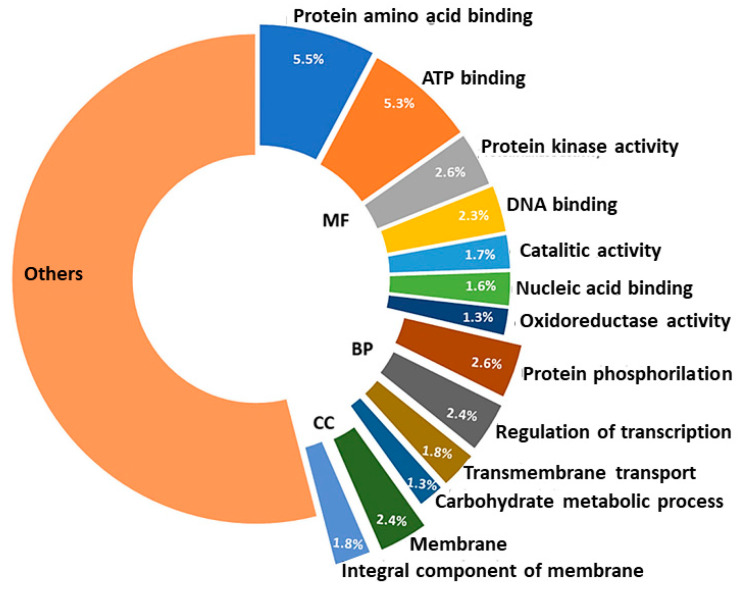
Thirteen main sub-GO categories of genes containing SSRs.

The GO categorisation of the genic SSR highlighted 1113 sub-categories of three main GO categories—Biological Process (BP), Molecular Function (MF), and Cellular Component (CC). Thirteen sub-GO categories represented ~33% of the identified entries (Figure 5). 

The MF sub-categories “protein amino acid binding” (GO:0005515) and “ATP binding” (GO:0005524) represented more than the 10% of the overall identified accessions. The occurrence of SSRs within specific gene functions has been previously observed [22,37,55,56,57], as well as the presence of SSRs in binding-associated genes, specifically in the 5′-UTR region [49]. Unexpectedly, only 2.4% of the SSRs identified fell among the “regulation of transcription” (GO:0006355) sub-GO category (BP) as the accumulation of microsatellites in transcription factors, and more in general in transcription regulation loci has been repeatedly reported in literature [22,37,55,56,57].

### 2.4. AnCorDB Construction, System Architecture, Features and Utility

**Figure 6:**
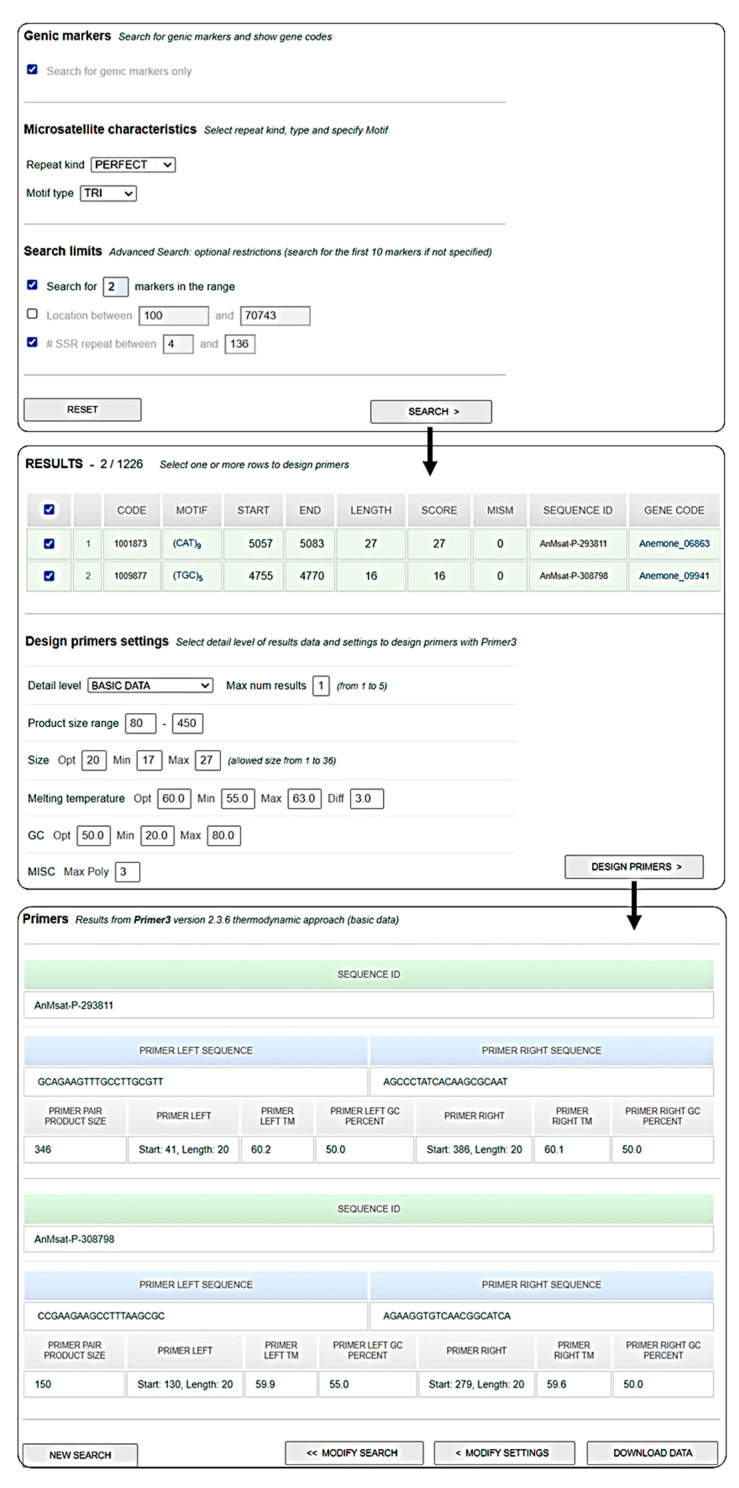
Example of SSR search and primer design at *AnCorDB*.

A public and searchable database of the microsatellites data reported in this paper was developed (*AnCorDB*—Available at www.anemone.unito.it, accessed on 9 March 2022). It offers similar features to the *CyMSatDB* [37] and the *EgMiDB* databases [38] and it can be used to retrieve SSRs based on either simple and complex searches. The database provides browsable access to all the SSRs identified in the poppy anemone genome. SSRs can be retrieved on the basis of simple characteristics, such as “SSR feature” (whole genomic or only genic SSR), “repeat kind” (perfect vs imperfect), or advanced characteristics, such as “motif type” (mono- to hexanucleotide), “specific motif sequence”, “repeat number”. Multiple parameters can be also combined to search for a specific set of SSRs as per user requirement, as researchers can limit the search via motif repetition and number of markers required (1–99). Scaffold position can be changed through a dedicated query. The output lists a wide range of information (SSR identifiers, scaffold number, motif type and length, genomic location—start and end position-, SSR length) including an optional download of the flanking sequences. Primer3 tool is implemented in the database, allowing primers design through the “Design Primers” button which directs the use to a list of up to five possible primer pairs, with their melting temperatures (Tm), their GC content, and the expected length of the amplicon. The obtained pairs of primers can be downloaded in MS-Excel format (Figure 6).

### 2.5. SSR Validation and Varietal Fingerprinting

A set of 150 microsatellite loci was selected as representative of the overall genome distribution of every class and motif, their primer pairs were designed, and they were PCR-validated. On the basis of the amplicon quality, 62 SSRs were selected for varietal fingerprinting of poppy anemone cultivars (Supplementary Materials). In some cases we detected a low efficiency of the primer design which could be attributed to the low coverage of our draft genome, leading to misassembly in the repetitive elements regions [58,59]. Nevertheless, the percentage of suitable primer pairs resulted in line with the one detected in previous SSR mining reports based on draft genome sequence obtained at low-coverage [60,61,62].

We tested the selected 62 SSR markers on eight commercial cultivars of which six were diploids and two tetraploids (see Section 3), representative of the phenotypic variability of the varieties marketed by Biancheri Creazioni. A total of 203 alleles were generated, with a mean of 3 (range 1–8) alleles per locus. The largest range in amplicon length detected was 199–604 bp, resulting from the amplification of *Ancor33*, a dinucleotide AT motif. In the 25.8% of the loci, the assay generated the amplicon predicted length, while in the 48.4% the amplicon was longer than expected and in the 25.8% shorter. Only three SSRs were monomorphic across the evaluated genotypes and thirty markers were nullallelic for at least one genotype. The polymorphism information content (PIC) values of the polymorphic SSRs varied from 0.13 to 0.85 (mean 0.52 ± 0.025). *AnCor49* had the highest PIC, and *AnCor71* the lowest (Supplementary Materials).

**Figure 7:**
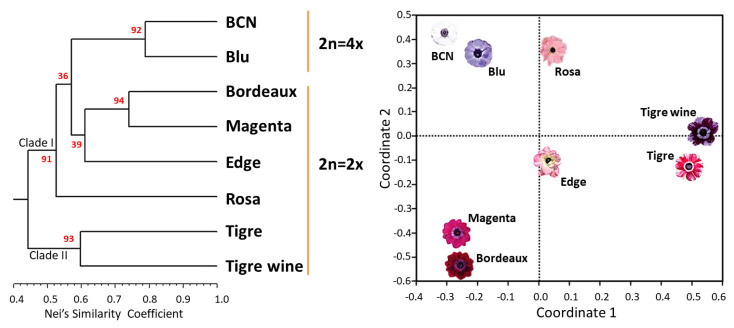
UPGMA dendrogram (**left**) and PCoA analysis of the eight varieties based on 62 microsatellite loci (**right**). Bootstrap values (%) are reported in red.

The scored allele peaks were used to elaborate a UPGMA-based dendrogram (Figure 7) which allowed the fingerprinting of each cultivar.

The detected genetic relationships among varieties was in accordance with their breeding origin (Biancheri, personal communication). Two major clades were identified, and supported with bootstrap values higher than 90. In Clade I, the two tetraploid cultivars (“BCN” and “Blu”) clustered with an average genetic similarity of 78% and a bootstrap probability of 92%, while among the other three cultivars, Edge resulted more genetically differentiated from “Bordeaux” and “Magenta”, which showed a genetic similarity of 74% and clustered with a bootstrap probability of 94%. In Clade II, “Tigre” and “Tigre Wine”, which in turn resulted highly differentiated, showed an average genetic similarity of 60%.

The first two axes of the PCoA scatter plot (Figure 7) explained 42 and 31% of the overall genetic variation respectively confirming the genetic relationships between the cultivars. Interestingly, the cultivars “Edge” and “Rosa”, although resulted genetically differenciated following UPGMA analyses, showed a common value for the first main coordinate of the PCoA.

Aiming at developing a fingerprint protocol for poppy anemone and identifing the minimum number of SSR loci needed to fully discriminate between the cultivars in study, we selected six SSRs. Five of them were selected on the basis of their PIC values, namely the dinucleotide SSRs *Ancor33*, -*36*, -*49*, -*59*, and -*83*, applied togheter with the tetranucleotide *Ancor177* (detailed information in Supplementary Materials). On the basis of the 6 SSR markers, we created a similarity matrix and correlation between this matrix and the one obtained using the whole data set indicated a good fit of the genetic relationships (r = 0.92) and made it possible to fingerprint each cultivar. This suggests their possible application as a valuable tool for varietal identification in the species.

### 2.6. Intra-Cultivar Variability Assessment

**Figure 8:**
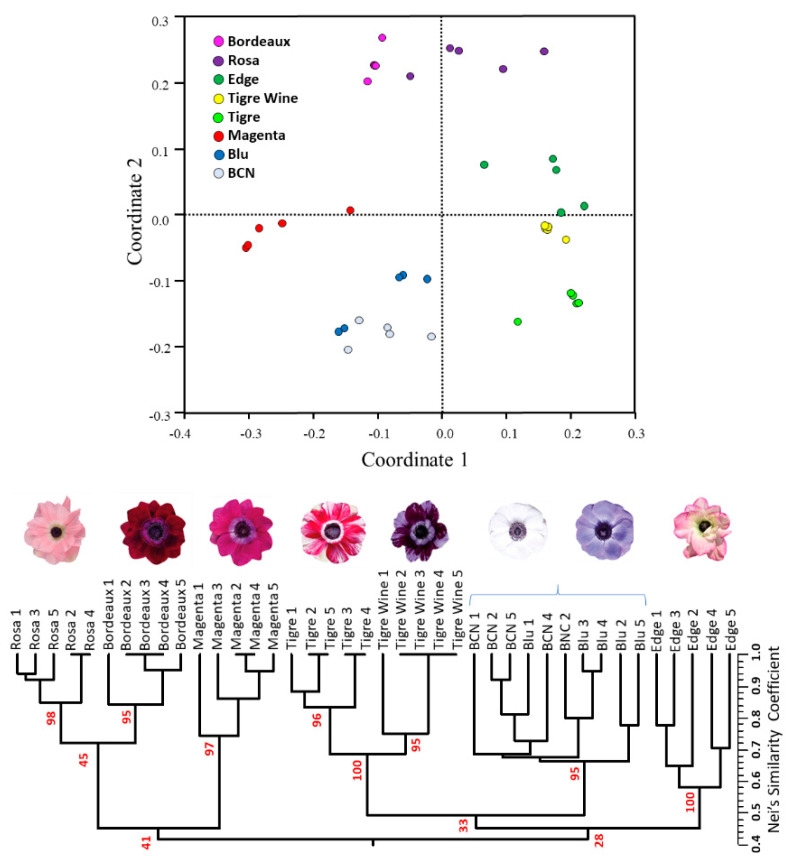
Dendrogram and PCoA obtained from UPGMA cluster analysis of five plants for each of the eight cultivars, based on six microsatellites (47 alleles). Bootstrap values (%) for the main nodes are reported in red.

In order to assess the intra-cultivar variability among the 8 cultivars in study and furtherly validate the newly developed SSRs, five plants per cultivar were genotyped using the previously described set of six microsatellites. Fixation index (F_IS_) values ranged from −0.68 to 0.79. As expected from selected genotypes obtained within breeding programs, most the loci showed significant deviation from HWE, with only one marker (*Ancor89*) showing no significant difference between expected (H_E_) and observed (H_O_) heterozygosity values (Supplementary Materials). The principal coordinate analysis and UPGMA dendrogram illustrate the genetic relationships between members of this extended germplasm panel (Figure 8).

PCoA axes 1 and 2 accounted for ~74% of the overall genetic variation, the former contributing ~48%, and the latter ~26%. As expected, the cultivar “Edge” and “Rosa” shared positive (or slightly negative) values for the first coordinate, together with “Tigre” and “Tigre Wine”. “Edge” showed the highest intra-cultivar variability, while “Bordeaux” the lowest (Figure 7). The UPGMA based on 62 and six SSRs in some cases provided different clustering among the cultivars under study. This is the case of the cultivars “Rosa” and “Bordeaux”, which appeared more genetically distant on the basis of 62 microsatellites (Figure 7), while closer on the basis of six SSRs (Figure 8) and with a bootstrap value as low as 45.

Each plant of the cultivars “Edge”, “Blu”, and “BCN” showed a unique fingerprinting, while in the other five, some plants shared common alleles. Despite the observed intra-cultivar genetic variability, the application of only six SSRs made it possible to clearly discriminate the diploid cultivars, each of them clustered with bootstrap support from 95 to 100, while no clear genetic differentiation between the tetraploid cultivars “BCN” and “Blu” was detectable, suggesting the application of additional specific markers for their fingerprinting. For this purpose, the sixty-two amplified markers were investigated, leading to the identification of five microsatellites each of which might be individually applied for “BCN” and “Blu” discrimination (Table 2). 

## 3. Materials and Methods

### 3.1. Draft Genome Sequencing, Assembly, and Annotation

Leaves of a haploid plant originated from the commercial line MISTRAL^®^ MAGENTA obtained through “in vitro” androgenesis by applying the regeneration protocol adapted by [5], were provided by Biancheri Creazioni (Camporosso (IM), Italy). Plant DNA Kit (E.Z.N.A.^®^) was used for the genomic DNA extraction following the manufacturer’s instructions. DNA quality was assessed through the NanoDrop™ 2000 spectrophotometer and the Qubit^®^ 2.0 Fluorometer was used for DNA quantification. One microgram of DNA was used for the construction of a 270 bp insertion library (Novogene, Hong Kong), which was sequenced using a NovaSeq Illumina platform (Illumina Inc., San Diego, CA, USA) with paired-end chemistry (2 × 150 bp). Raw reads were cleaned with Scythe (v0.994, https://github.com/vsbuffalo/scythe, accessed on 2 January 2022) for removing contaminant residual adapters and Sickle (v1.33, https://github.com/najoshi/sickle, accessed on 2 January 2022)) for removing reads with poor quality ends (Q  <  30). De novo assembly was performed with standard parameters using the MEGAHIT assembler ([63]; https://github.com/voutcn/megahit, accessed on 2 January 2022)), an ultra-fast and memory-efficient NGS assembler based on succinct de Bruijn graphs that can be applied both for metagenomics and single genome assembly. The quality of the genome assembly (e.g.,: N50, scaffolds/scaffolds number/size/length, genome length) was assessed using the perl script Assemblathon_stats.pl ([64]; https://github.com/ucdavis-bioinformatics/assemblathon2-analysis, accessed on 2 January 2022)). Cleaned reads were then used for k-mer-based genome size estimation using the jelly-bean software and applying the formula Genome Size = 19-mers count/peak position of the number of times each 19-mer occurs (see Supplementary Materials).

The assembled draft genome was pre-masked using RepeatMasker v4.1.0 [65] with a de novo approach. A species-specific repeats library was constructed following the Repeat Library Construction Advanced pipeline ([66]-http://weatherby.genetics.utah.edu/MAKER/wiki/index.php/Repeat_Library_Construction-Advanced, accessed on 2 January 2022)) which requires the use of mite hunter, LTRdigest, LTR_harvest (available in genome tools, v1.5.10), and Repeatmodeler v1.0.11. The new library was then combined with Repbase-viridiplantae to identify transposable elements (TEs). TEs were classified into two main classes: Class I (retrotransposon elements) and Class II (DNA transposons). Gene prediction was performed using Maker-P v2.31.08. Augustus v3.3.2 ([67]) Hidden Markov Models, and SNAP ([68]) gene prediction algorithms were combined with transcripts and protein alignments as evidence to support the prediction. All predicted gene models were filtered and only the ones with an AED ≤  0.4 were maintained. AED measures the concordance of a gene predicted with aligned transcripts, mRNA-seq, and protein homology data. AED scores range from 0 and 1, where 0 indicates perfect concordance between evidence and gene prediction, while 1 absence of concordance. To measure the quality and completeness of the predicted proteomes, a quantitative assessment was carried out based on evolutionary informed expectations of gene content known as Benchmarking Universal Single-Copy Orthologs (BUSCO v3.0.2., Embryophyta odb 10—[69]). The sequences of the predicted proteins were also noted using InterproScan5 ([70]) compared to all the available databases (ProSitePro les-20.119—[71], PANTHER-10.0—[72], Coils-2.2.1—[73], PIRSF-3.01—[74], Hamap-201511.02—[75], Pfam-29.0—[76], ProSitePatterns—20.119—[71], SUPERFAMILY-1.75—[77], ProDom-2006.1—[78], SMART-7.1—[79], Gene3D-3.5.0—[80], and TIGRFAM-15.0—[81]). Then, GOfeat ([82]) was used to identify the enrichment of GO terms for specific gene clusters.

### 3.2. SSR-Mining

The un-masked draft assembly of the *A. coronaria* L. genome was used for SSR mining. Scaffolds were chopped into manageable pieces using SciRoKo tool ([83]—v3.4; https://kofler.or.at/bioinformatics/SciRoKo, accessed on 2 January 2022)), and perfect, compound, and imperfect SSRs were identified in silico using SciRoKo and the misa.pl pipeline (https://github.com/cfljam/SSR_marker_design, accessed on 2 January 2022)). A minimum of four repetitions together with a minimum length of 15 nt were requested. Any sequence was considered as a perfect SSR when a motif was repeated at least fifteen times (1 nt motif), eight times (2 nt), five times (3 nt), or four times (4–6 nt), allowing for only one mismatch. For compound repeats, the maximum default interruption (spacer) length was set at 100 bp. The coordinates (start/end position) of each SSR were matched with those of the gene space using Bedtools intersect (using the default parameters) with -loj (left outer join) option: where the overlap comprised at least 1 nt, the repeat was designated as a genic SSR. A GO categorization of the three main GO categories—“biological processes” (BP), “molecular functions” (MF), and “cellular components” (CC)—were applied to genes carrying at least one SSR. 

### 3.3. AnCorDB, an SSR Database for Poppy Anemone

The *Anemone coronaria* Microsatellite DataBase (*AnCorDB*; www.anemone.unito.it, accessed on 9 March 2022)) was developed to provide browsable access to the SSR data. This web application, based on a LAMP stack, comprises a client tier (client browser), a middle tier (Apache web server with PHP interpreter), and a database tier (MySQL DBMS). A user-friendly interface was developed using PHP, which is an open-source server-side scripting language. The set of in silico detected SSRs were stored in the MySQL database, using PHP scripts to parse the text file from SciRoKo. User need-based customized queries can be generated from the web interface and allow users to search the microsatellite marker information in MySQL database. A stand-alone version of Primer3 has been also provided to design primer pairs for any given SSR: its output lists alternative sets of primer pairs, and the characteristics of the expected amplicon.

### 3.4. Marker Validation

One hundred and fifty microsatellites were selected among the ones with a number of repetitions between 20 and 30, in line with the overall genome representation of every class and motif. Specifically, di- and tri-nucleotides were selected in the interval ranging from 25 and 30 motif repetitions, while this threshold was lowered to the interval between 20 and 30 motif repetitions for the other classes of microsatellites. These parameters were applied with the aim of obtaining the highest potential polymorphism rate of the selected markers. The primer pairs obtained from the database were used for the DNA amplification of eight *A. coronaria* cultivars representative of the phenotypic variability of the ones marketed by Biancheri Creazioni. Among them, six cultivars were diploid (“Bordeaux”, “Edge”, “Magenta”, “Rosa”, “Tigre”, and “Tigre Wine”), while two were tetraploid (“BCN” and “BLU”). The following touchdown PCR protocol was applied: 94 °C for 5 min followed by 13 touchdown cycles with denaturation step at 94 °C for 30 s, a step at 60 °C for 30 s decreasing the annealing temperature of 0.38 °C every cycle, and lastly extension step at 72 °C for 30 s. At last, 35 cycles at 94 °C for 30 s (denaturation), 55 °C for 30 s (annealing), and 72 °C for 30 s (extension), and a final extension cycle at 72 °C for 5 min. PCR products were separated using a 2% agarose gel to check their occurred amplification. 

### 3.5. SSR Fingerprinting and Intra-Cultivar Variation Assessment

A subset of 62 SSRs (Supplementary Materials) were further analyzed through capillary sequencing (ABI PRISM^®^ 310, Applied Biosystems™). M13-Tailed Forward primers were designed for each microsatellite and applied in a three-primers unbalanced PCR reaction with a fluorescent-labelled M13 primer ([84]). PCR was carried out in a final volume of 20 μL containing: 4 μL of 5× GoTaq Colorless Buffer (GoTaq^®^ DNA Polymerase, Promega), 1 μL of MgCl_2 (25 mM), 0.4 μL of dNTPs (10 mM), 3 μL of DNA template (5 ng/μL), 1 μL of Reverse and M13-labeled primer (10 μM), 0.2 μL of Forward-M13 Tailed primer (10 μM), and 9.2 μL of ultrapure water. In each reaction, 1 μL of amplification product was pooled with other three products labelled with different fluorophores (FAM, VIC, NED, and PET) and purified using the PEG-precipitation method described by [85]. Multiplex genotyping reactions were carried out in ABI PRISM^®^ 310 according to the GeneScan^®^ Reference Guide (Applied Biosystems™). Results were visualized using Peak Scanner™ Software v1.0 (Applied Biosystems™) and for each microsatellite the amplicons’ length was scored. A binary matrix was generated by scoring the band presence (1) and absence (0), which was used to compute pairwise similarity coefficients [86] and then to construct a UPGMA-based dendrogram [87] with 1000 bootstraps. Principal coordinate analysis (PCoA) was also performed for displaying the multi-dimensional relationship between genotypes, and the two axes were plotted graphically, according to the extracted eigenvectors. All analyses were performed using the NTSYS software package v2.10 [88] and Past 4.09 software [89]. The polymorphic information content (PIC) was calculated for each locus as described by [90] and used for selecting the most informative SSRs and identify the lowest number of loci needed for fingerprinting each of the cultivar in study. Mantel test [91] was performed to establish correlations between the similarity matrices generated by the most informative SSRs with the one generated from the complete data set. An intra-cultivar variability assessment was also performed by applying the most informative SSR loci on five plants belonging to each of the eight cultivars. PCR reactions, capillary sequencing, UPGMA-based dendrogram, and PCoA analysis were performed as described above. Calculations of observed (H_O_) and expected (H_E_) heterozygosity and Wright’s fixation index (F_IS_) were estimated with the program identity 1.0 [92]. Exact tests of Hardy–Weinberg equilibrium (HWE) were made by means of the software genepop 3.4 [93].

## 4. Conclusions

The development of a draft genome assembly of *Anemone coronaria* L. represents the first step toward genomic studies in poppy anemone. Its availability made it possible to identify a wide set of SSR markers and release the comprehensive microsatellite database *AnCorDB* (www.anemone.unito.it). The latter contains a full set of information regarding both genic and non-genic, perfect and imperfect SSR loci. Its intuitive web interface and its customized primer design offer a highly flexible tool to the scientific community and breeders, exploitable for genetic as well as phylogenetic studies. Our results also demonstrated that the application of a limited number of SSRs might be suitable for varietal discrimination and may contribute to solve Breeding Rights disputes. 

## Figures and Tables

**Table 1:** Microsatellite motifs distribution across the assembled genome. Perfect (including compound) and imperfect SSR are reported.

		Mono-	Di-	Tri-	Tetra-	Penta-	Hexa-	Total/Mean
Perfect SSR	Types	2	4	10	32	91	304	443
	Count	13,475	241,693	95,326	27,203	10,805	13,320	401,822
	%	3.4	60.2	23.7	6.8	2.7	3.3	100
	Density (SSR/Mbp)	2.2	39.41	15.54	4.44	1.76	2.17	65.52
	Cumulative (Mbp)	0.05	1.94	1.14	0.43	0.21	0.32	4.11
	Cumulative (%)	0.08%	47.20%	27.74%	10.46%	5.11%	7.79%	100%
	Mean Repeat Number	22.7	11.3	6.9	5.2	5.0	6.8	57.9
Imperfect SSR	Count	2823	111,281	38,183	10,719	12,920	13,061	188,987
	%	1.49%	58.88%	20.20%	5.67%	6.84%	6.91%	100%
	Density (SSR/Mbp)	0.46	18.14	6.23	2.15	2.11	2.13	31.22

**Table 2:** List and primer sequences of the six candidate markers for cultivar discrimination analyses between BCN and Blu.

				Alleles (bp)	
SSR	SSR Type	Motif	N° of Repeats	BCN	BLU
AnCor49	Di	AT	26	450; 468	435
AnCor74	Di	GT	30	501; 530	529; 539
AnCor87	Di	TC	29	271	262; 275
AnCor115	Tri	AAC	28	518	528
AnCor132	Tri	AAC	27	533; 589	536; 572
AnCor139	Tri	AAG	26	501	504; 512

## Data Availability

Sequencing data used in this study are openly available in the NCBI database (PRJNA808392).

## Supplementary Materials

The following supporting information can be downloaded at: www.mdpi.com/article/10.3390/ijms23063126/s1.
